# Social prescription for the elderly: a community-based scoping review

**DOI:** 10.1017/S1463423624000410

**Published:** 2024-10-17

**Authors:** Rute Sadio, Adriana Henriques, Paulo Nogueira, Andreia Costa

**Affiliations:** 1 Nursing Research, Innovation and Development Centre of Lisbon (CIDNUR), Nursing School of Lisbon (ESEL), Lisbon, Portugal; 2 ULSAC - Unidade Local de Saúde do, Alentejo Central, UCSP Estremoz, Estremoz, Portugal; 3 Instituto de Saúde Ambiental (ISAMB), Faculdade de Medicina, Universidade de Lisboa, Lisboa, Portugal; 4 Laboratório para a Sustentabilidade do Uso da Terra e dos Serviços dos Ecossistemas – TERRA, Lisbon, Portugal

**Keywords:** community reference, elderly person, primary health care, social prescription

## Abstract

**Aim:**

This scoping review aimed to identify the social prescription activities that exist for the elderly in a community context.

**Background:**

The increase in population ageing imposes the need to implement specific actions that guarantee elderly people the possibility of experiencing this phase with quality. The pandemic significantly exacerbated the needs of the elderly, leading to, regarding the loss of functional capacity, quality of life, well-being, mental health, and increased loneliness. Social prescription emerges as an innovative and non-clinical strategy, being a personalized approach that focuses on individual needs and objectives (Islam, 2020). By referring primary health care users to resources available in the community, obtaining non-medical support that can be used in conjunction with, or instead of, existing medical treatments (Chng *et al.*, 2021).

**Methods:**

A scoping review was conducted based on preferred reporting items for systematic reviews and meta-analyses, extension for scoping reviews (PRISMA-ScR). Searches were performed in electronic databases for potential studies: Scopus, PubMed, Medline, and Psychology and Behavioral Sciences Collection. Studies were included if they: (1) addressed social prescription interventions; (2) were community based; and (3) included elderly participants. Data extraction followed predefined criteria.

**Findings:**

Of a total of 865 articles identified, nine were selected. The social prescription activities identified fall into eight main domains: arts, personal development, social interaction, physical activity, gardening, cultural activities, religious activities, and technological activities. The interventions resulted in improved well-being, enhanced quality of life, health promotion, and reduced isolation and loneliness. Social prescription, while innovative, is still an evolving intervention, which can respond to the needs of the elderly population, given the range of activities that may exist in the community. Primary care professionals must develop these interventions, establish a link between health and the community, respond to these needs, and promote healthy ageing.

## Introduction

There is a global demographic change, with the population ageing at an increasingly rapid pace. The number of people aged 60 or over in the world is expected to increase from 1.1 to 1.4 billion by 2030. Adding that by 2050, the global population of elderly people will double (World Health Organization, 2023). This trend is particularly pronounced in Portugal, which is projected to be the most-aged country in European Union by 2050 (United Nations Department of Economic and Social Affairs, (2019)). The National Institute of Statistics (INE, 2020) estimates that by 2080, the ageing index in Portugal will nearly double, with an estimated 300 elderly individuals for every 100 young people.

The increase in life expectancy worldwide has presents a continuous challenge to society, particularly in terms of health issues associated with ageing. While the increase in life expectancy is a significant achievement, it does not intrinsically imply an improvement in the quality of life. Ageing presents challenges to society and health professionals, especially those in primary health care, necessitating specific actions to ensure a quality life for the elderly (Peixoto *et al.*, 2017).

With ageing, there is an emergence of comorbidities and physiological consequences, which are often associated with lifestyle factors such as poor nutrition and a sedentary lifestyle. These factors impact the ability of the elderly to maintain their independence (Benedetti *et al.*, 2008; Morley & Silver, 1995).

In Portugal, the increase in the dependency rate of elderly people demonstrates the challenges facing the current healthcare system. In 2019, the elderly dependency index was 34.5 projections from the National Institute of Statistics (INE) indicate that in 2065 this indicator will increase between 66 and 76 (INE, 2020), leading to a major drop in the sustainability index potential, rising in 2020 from 285 to 2065 with a value of 141 (INE, 2020). It was also found that between 2001 and 2011, the proportion of single-person families with people aged 65 years or over increased in all regions of Portugal, being highest in the Centre, Lisbon, Alentejo, and Algarve regions. (INE, 2014). This fact leads consecutively to institutionalization, being able accelerate and accentuate the decline in the physical and cognitive functions of elderly people with dependency (ONLCP, 2020). Elderly people living in rural areas often face difficulties, such as access to health and social services, and may face risks of worse health status, lack of transport services, and opportunities for social participation, creating inequalities compared to people living in rural areas urban sites (Comissão económica, 2017).

Therefore, it is necessary to create better opportunities for healthy ageing and the well-being of rural populations.

The COVID-19 pandemic exacerbated these factors leading to social isolation loneliness and mental issues in the elderly (Kyriazis *et al.*, 2021; Lebrasseur *et al.*, 2021). This pandemic has worsened health inequalities and the important role of systems in addressing the social determinants of health (Morse *et al.*, 2022). The effects of the COVID-19 pandemic affected older people on a physical, psychological, and social level, and there was a decrease in social life and fewer face-to-face social interactions were reported during the COVID-19 pandemic. There was a reduction in quality of life and an increase in depression, as well as difficulties in accessing services, namely health, sleep disorders, and reduced physical activity, leading to a change in their physical capabilities, with an increase in dependence (Lebrasseur *et al.*, 2021; Ömer *et al.*, 2021).

Therefore, it becomes imperative that we adopt policies and programs, where health professionals focus their practice on strategies to address these needs of the elderly, so that they remain healthy, active, and independent, thus promoting healthy ageing, reducing the proportion of elderly people below the disability threshold and contributing to the sustainability of health systems (Graybill *et al.*, 2014). The needs of the ageing process refer the need for non-clinical interventions, where traditional health structures do not provide a correct answer in order to affect the health and well-being of the person (MacLeod *et al.*, 2018).

In response to these challenges, social prescribing has emerged as a complex health model, a response to people’s non-clinical needs, assuming a potential role in effectively combating social determinants of health, as well as exacerbating pre-existing diseases (NHS England and NHS Improvement, 2020). Social prescribing is a strategy that can be used by primary healthcare professionals to prescribe activities developed by the tertiary sector, connecting users to existing resources and activities in the community to improve their health and well-being (Pescheny *et al.*, 2018). It is considered a salutogenic model, whereby each person, community, or organization has health assets through which they can deal with problems. It is intention is to offer people a holistic approach to care, helping to address the underlying causes of their health rather than simply treating their symptoms (Boydell, 2020). The operationalization of the social prescription can be done in different ways of referral, the primary health care professional, makes the signage providing information about a community program; or moving the person directly from primary care to a community program; or referral by connecting the person to a link-worker, who helps the person set goals and encourages them to achieve them, being able to monitor social prescription activity (Husk *et al.*, 2020).

Social prescription is considered a future direction, the key to providing community care (Mendes, 2021; Smith *et al.*, 2019), becoming the appropriate, innovative, specific response in health promotion that responds to various problems, and needs felt by many elderly people. Social prescription provides an individualized approach, centred on the needs and goals of the person, creating goals together, based on their strengths and resources available in their communities (Islam, 2020). It is seen as a preventive measure, facilitating individuals’ access to services and activities that may reduce the need for medical intervention in the future, facilitate the reduction of inequalities and the empowerment of the individual in the community (Calaf and González-Vianab, 2021). It aims to reduce dependency on traditional health services, such as medication and hospital visits, addressing the social and emotional influences that may be affecting the patient’s health.

**Figure 1.**
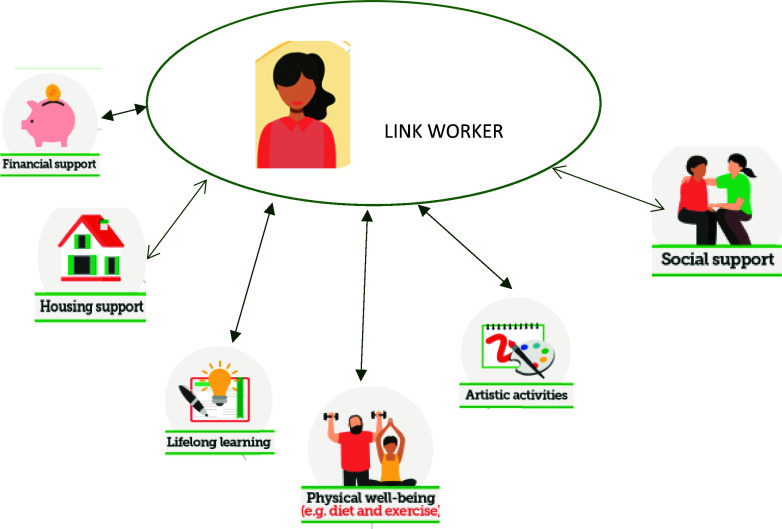
Link worker and connection to community resources, adapted from (World Health Organisation, 2022).

Link workers must listen carefully to the needs of the elderly person, working together, with a person-centred plan that connects them to existing community resources, such as volunteering opportunities, social support, artistic activities, physical well-being, lifelong learning, housing support, financial support (World Health Organisation, 2022), as represented in Figure 1. According to the National Health Service the social prescriber can link you to healthy lifestyles advice; gardening and horticulture; education and learning opportunities; benefits and money matters; befriending, talking therapies, and support groups; arts, music, dance, sport plus other groups and activities; employment, training and volunteering opportunities; veterans ‘support; and access to specialist services and support. This way, the social prescription activities include artistic activities, gardening, physical activity, volunteer roles, cooking classes, group learning, social activities, classes gymnastics, local support groups, and healthy eating (Chatterjee *et al.*, 2018). These activities aim to reduce reliance on traditional health services, such as medication and hospital visits, by addressing the social and emotional influences that may be affecting the patient’s health.

In this way, the social prescription in primary health care provides an effective and efficient response to all these points identified above, with the need for the multidisciplinary health team to act, verifying the need to identify and map which interventions social prescription that can be applied by the primary care professional in a community context. Understanding the offer that community organizations and local authorities can offer from support activities, cultural, educational, or environmental activities (Morton *et al.*, 2015), which correspond to the needs presented by the user.

Given the potential of social prescription and the current gaps in understanding its application for the elderly in community settings, this scoping review aims to identify the existing social prescription activities for the elderly in a community context.

Given the need for non-clinical interventions to address the needs of the ageing population, it becomes imperative to adopt policies and programs that focus on these needs. As this approach is still innovative and underutilized in Portugal, it can make a significant difference (Mendes, 2021). Furthermore, it aligns with the Sustainable Development Goals included in the 2030 Agenda for Sustainable Development, specifically ensuring healthy lives and promoting well-being at all ages (World Health Organization, 2015).

## Methods

The scoping review was conducted based on the five-step framework by (Arksey & O’Malley, 2005), with enhancements from (Levac *et al.*, 2010), and adhered to the PRISMA guidelines (Page *et al.*, 2021; Tricco *et al.*, 2018) to ensure reliability and replicability. This framework consists of five stages: (a) identification of the research question; (b) identifying relevant studies or search strategy; (c) study selection; (d) data charting and assessing the quality of studies included and (e) collating, summarizing, and reporting the results (Arksey & O’Malley, 2005).

### Identification of the research question

The review was guided by the following research question: “What social prescription interventions exist for the elderly in a community context?”

This review is different from other reviews on social prescribing that have already been carried out, as these mainly focus on the effectiveness of social prescribing interventions, while in this case a mapping of interventions is carried out.

### Identification of relevant studies

Firstly, a search was carried out in several databases to identify articles on the topic, obtaining the keywords to construct a search strategy that was as complete as possible in the databases that were intended. Potential studies were identified through searches in four electronic databases: Scopus, PubMed, Medline, and Psychology and Behavioral Sciences Collection. Preliminary searches were conducted between June and December 2022, with final search performed on 3 January 2023.

The search strategy combined three main conceptual terms and equivalent terms, equally across all databases in order to cover all articles that could be relevant, as can be seen in Table 1. Boolean operators (AND, OR) were used in the summary: ‘elderly’, ‘social prescription’, and ‘primary health care’. For the PubMed database, Medical Subject Headings (MeSH) terms were used where possible. Norestrictions were placed on the publication date or language of the articles.

**Table 1.** Scoping review’s research strategy

Quest	Search	Scopus	PubMed	Psychology and behavioral sciences collection
#1 *Population*	“older people” OR “aging” OR “aged” OR “elderly” OR “older adults” OR “geriatric” OR “geriatrics” OR “senior” OR “seniors” OR “aged 65” OR “over aged 65”	7 071 373	1 769 208	75 676
#2 *Concept*	“social prescription” OR “social prescribing” OR “community prescribing” OR “community prescription” OR “non-medical referral” OR “arts on prescription” OR “books on prescription” OR “exercise on prescription” OR “education on prescription” OR “museums on prescription” OR “music on prescription” OR “dance on prescription”	697	10 326	69
#3 *Context*	“community health nursing” OR “primary care” OR “community care” OR “primary healthcare” OR “health care delivery” OR “community activities” OR “primary care nursing” OR “community intervention”	484 001	111 191	19 590
#4	#1 AND #2 AND #3	73	756	1

After eliminating articles that were not relevant to the objectives of this study, the reference lists of articles that were selected were checked to search for other significant sources, ensuring greater confidence in the identification and review of sources.

### Study selection

**Figure 2.**
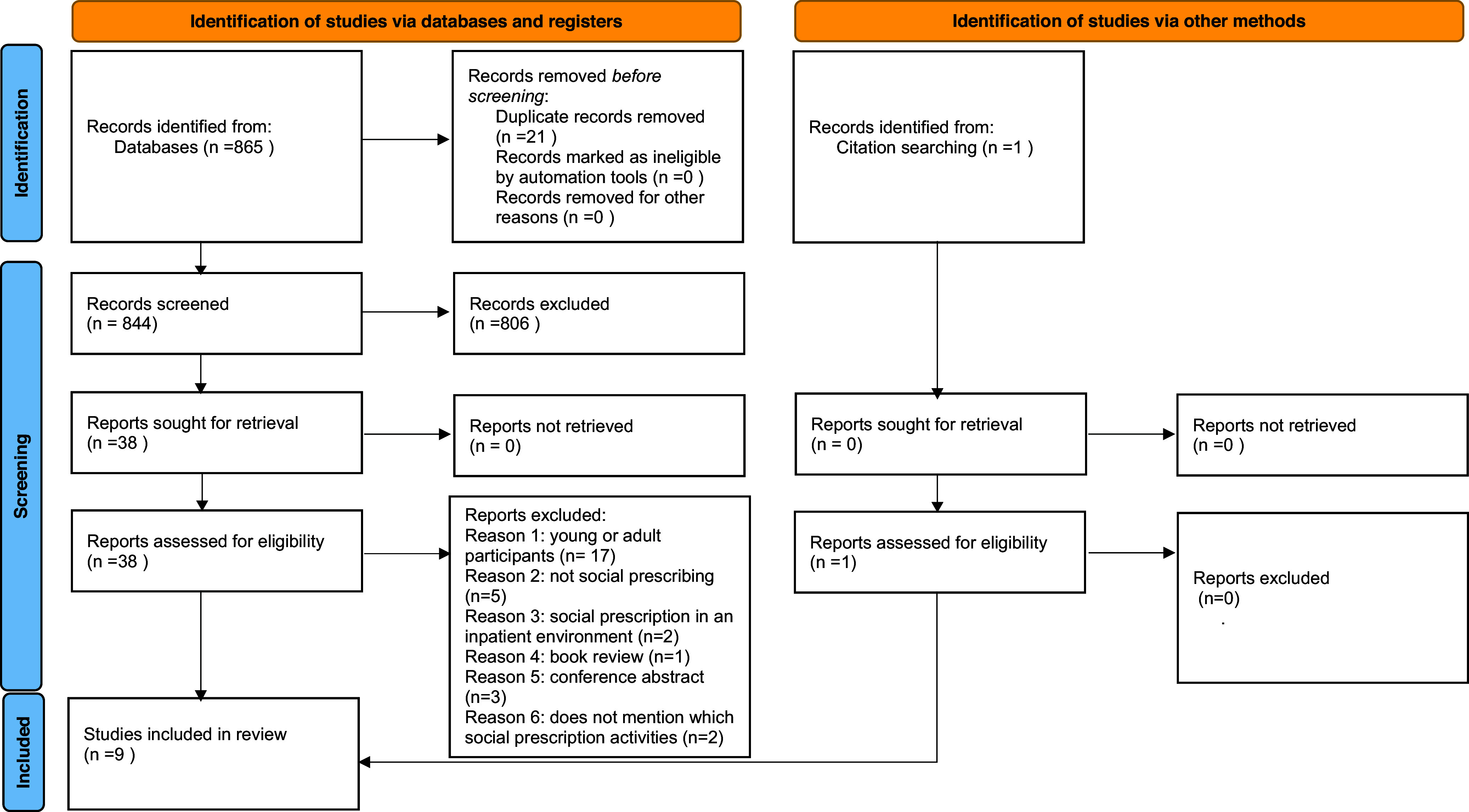
The PRISMA 2020 statement: an updated guideline for reporting systematic reviews. From: Page MJ, McKenzie JE, Bossuyt PM, Boutron I, Hoffmann TC, Mulrow CD, *et al.* BMJ 2021;372:n71. doi: 10.1136/bmj.n71. For more information, visit: http://www.prisma-statement.org/.

Duplicate articles were removed using Covidence software. An initial selection by title and abstract was completed by two members of the research team, and the title and overview of the gray literature were selected, with the purpose of checking whether they were relevant to the research objective. The selected articles were then read in full, by all team members, for final inclusion. Articles were included if they cumulatively met the following criteria: (1) they discussed a social prescription intervention; (2) the intervention was implemented in a community setting; and (3) the intervention targeted elderly individuals. Studies were excluded if they did not include elderly individuals or if the social prescription intervention was not explicitly described. The PRISMA 2020 flowchart was used to describe the study selection process and consequently their screening (Figure 2).

### Data charting

Two researchers independently evaluated the full text of the included articles.

These two researchers extracted the data, using a synoptic table with pre-defined variables, such as, author, year, country, region, study design, target population, social context, and social prescribing concept (Table 1).

In the presence of doubts or ambivalences, these were resolved in discussion with the other two researchers and were resolved unanimously.

### Collating, summarizing, and reporting the results

The results were synthesized narratively to address the research question.

Data analysis was an interactive process in which all researchers participated together, so that all data was obtained consistently in this research. Two stages of data analysis are included: carrying out a descriptive numerical analysis and a thematic analysis (Levac *et al.*, 2010). The first stage consisted of describing the number of research, study designs, year of publication, study population, and study location and the second stage followed qualitative data analysis techniques, also considering the recommendations of the World Health Organization: overview of participants, domains of social prescribing (SP) activities, duration of SP interventions, instruments used, and main outcomes (Table 2).

**Table 2.** Overview of participants, domains of social prescribing (SP) activities, duration of SP interventions, instruments used, and principal outcomes

Author, year	Sample and age	Social prescribing activity (domains)	Social prescribing intervention	Outcomes (instruments)	Results
Loftus *et al.,* 2017	28 users, 5men and 23 women; over 65 years old, mean age 72.1.	Social Clubs, social interaction, arts program activities, craft activities, computer courses, fitness activities, fall prevention and counseling classes, crochet activities and personal development.	Intervention: 12 week programmes. Post-intervention assessment:12 week, 6–12 months later.	Number of visits to the family doctor, home visits, phone calls, impact on polypharmacy.	Increased self-esteem and well-being of users, without evidence of fewer visits to the family doctor or decrease in medication intake. It contributes to less isolation, lessening the impact of chronic illness and improving emotional well-being.
Thomson *et al.,* 2018	115 participants, 63% women; ages 65–94	Interactive museum tours, including lectures, behind-the-scenes tours, manipulation of objects and artistic activities inspired by the exhibitions.	Intervention: 12 programmes of 10 weekly Post-intervention assessment: start-programme, mid-programme e end-programme.	MwM-AO; interviews and weekly diaries made by the participants.	Improved psychological well-being over time. The study participants experienced a sense of privilege and appreciation of the opportunity to interact.
Kim *et al.*, 2021	10 participants, 100% women; with a mean age of 82 years.	The musical interventions, self-help group and gardening.	Intervention: 1 time a week during approximately 10 weeks. Post-intervention assessment: The survey was administered twice, before and after the interventions.	Geriatric depression scale (GDS); Geriatric Depression Scale-Korean Version (GDS-K); UCLA Loneliness Scale; Korean Adaptation of the General Self-Efficacy Scale; Korean translation of the Rosenberg Self-Esteem Scale.	Reducing depression and loneliness in the elderly. Health promotion in which self-esteem is positively influenced, as well as greater social participation of the elderly.
Clifford *et al.,* 2022	Between 10 and 12 seniors per group; 65 years or older.	Programme the music and dance	Intervention: 1.5-hour music and dance session per week for 12 weeks, along with a 1-hour music and movement program Post-intervention assessment: unrealized	Physical performance measures (Timed Up and Go Test (TUG); TUG-Cognitive; The 30 Second Sit to Stand Test; Single Leg Stance Test; Short physical performance battery (SPPB)); Self-report physical activity (Incidental and planned exercise questionnaire (IPEQ)); Loneliness (UCLA Loneliness Scale); Social Isolation (Berkman-Syme Social Network Index (SNI)); Cognition (The Trail Making Test (TMT)); Quality of Life and wellbeing and mood (The EQ-5D-5L; ICEpop CAPability measure for Older people (ICECAP-O); The 5-item Geriatric Depression Scale (GDS-5)); Qualitative evalutions (Focus groups and semi-structured)	No reported
Howarth *et al.,* 2020	47 people; ages between 30 and 85 years old, mostly over 60 years old.	Wellbeing Garden - Gardening activities	Intervention: 12-week pilot programme Post-intervention assessment: The survey was administered twice, before and after the interventions.	Warwick-Edinburgh Mental Well-being Scale (SWEMWBS); focus groups	Improved mental well-being, confidence and reduced social isolation.
Elston *et al.,* 2019	26 participants; + 50 years, mean age 79.6 years	Resilience training, emotional support and counseling and practical assistance in reaching local health, social and economic services.	Intervention: 12-week program Post-intervention assessment: The survey was administered twice, before and after the interventions.	Well-being Star; Patient Activation Measure (PAM); Warwick-Edinburgh Mental Health and Well-being Scale (WEMWBS); Rockwood Clinical Frailty Scale (RCFS).	Achieving significant improvement in health and well-being.
Kiely *et al.,* 2021	12 participants with a mean age of 63 years	The intervention involved follow-up phone calls or accompanying the person in community activities. Chronic disease self-management courses, addiction services and exercise classes. “Support was classified into instrumental (doing things for patients), informational (passing on information), emotional (listening and supporting) or appraisal (helping people to assess situations and make plans)” pág.3	Intervention: over a 6-week period Post-intervention assessment: The survey was administered twice, before and after the interventions.	EQ-5D-5L18; Hospital Anxiety and Depression Scale; Frenchay Activity Index; Patient Activation Measure; Multimorbidity Burden of Treatment Questionnaire; Social Connectedness Scale; Structured interviews.	The patients in this pilot, presented better health related quality of life, any less depression and anxiety and smaller more likely to live alone.
Moffatt *et al.,* 2017	30 adults, 14 women and 16 men, aged 40–74 (mean age 62)	Services that participants reported being linked into were as follows: physical activity/fitness, weight management, healthy eating, long term condition management, welfare rights services, community activities (e.g. choir, swimming, fishing, photography) and other voluntary organisations.	Intervention: between four and fourteen months. Post-intervention assessment: the interviews took place during the time of the intervention	Semi-structured interviews.	The intervention engendered feelings of control and self-confidence, reduced social isolation and had a positive impact on healthrelated behaviours.
Munford *et al.*, 2020	3.686 ranging from 65 to 69 years - the reference age group - up to 85 or older	Participation in group for elderly or older people, education, arts, music or singing group, religious group or church organisation, charity, voluntary or community group, social club, sports club, gym, exercise, or dance group.	Intervention: Community asset participation over time eighteen months Post-intervention assessment: 6-month, 12-month and 18-month.	Euro-QoL 5D-5L; costs of formal healthcare services and net social benefit	Participants reported significantly higher quality of life on all domains – environmental, psychological, physical, and social.

The content analysis method was applied to analyze the data and a narrative synthesis of relevant evidence to meet the objectives of the review.

## Results

Our initial search yielded 865 studies, out of which nine were selected for final analysis. The overview of the selected articles is in Table 3.

**Table 3.** Social prescribing studies characteristics

Author, year	Country, region	Study design	Target population	Social context	Social prescribing concept
Loftus *et al.,* 2017	UK; Northern Ireland	Observational non-controlled before-and-after study	Users with chronic conditions, frequent GP visits or taking multiple medications.	Urban community attending primary health care.	Health professionals (family doctors, social scientists and other professionals) work with users by selecting and referring them to existing social activities in the community to achieve healthy behaviors.
Thomson *et al.,* 2018	UK; Central London and Kent	Randomized controlled trial	Participants were people of various ethnicities, vulnerable, at risk of loneliness and social isolation	No reported	A local employee or link-worker who is in a primary health care organization or a third sector organization refers people and monitors community interventions according to the local agenda, being patient centred.
Kim *et al.,* 2021	South Korea; Wonju	Randomized controlled trial	Elderly people	Rural areas, during the COVID-19 pandemic.	Patients are diagnosed by the primary care physician and given a personalized social prescription, referral, supported by a link-worker, linking patients with nonmedical community services.
Clifford *et al.,* 2022	UK; West Central Ireland	Pragmatic cluster-randomised controlled feasibility trial	In order to participate, participants will have to be able to walk 3 meters, being able to use a walking aid and understand English or have someone who can translate so that the person can follow the instructions.	Participants residing in the community in the mid-west of Ireland, regions across three counties (Limerick - second highest level of total income per person in the State and has the lowest number of recreational facilities nationally, Clare - 65% of people in this county live in rural areas and Tipperary- most populated rural counties in Ireland)	Social prescription is seen as a community-based project using community resources. Referencing for social prescribing can be done by public health nurses, general practitioners, physiotherapists and occupational therapists. There may be publicity of activities with announcements in community areas or local newspapers.
Howarth *et al.,* 2020	USA; Bridgewater	Observational non-controlled before-and-after study	People who need to reduce anxiety, build confidence and improve physical or mental well-being.	Not reported	Social prescribing is seen as a non-medical asset-based process that connects the community based on a person’s individual preferences to improve their health and well-being. Community nurses in the locality forward patients residents an comunidade to the link worker.
Elston *et al.,* 2019	England; Devon	Observational non-controlled before-and-after study	Older adults in the community with health needs complex (≥2 long-term conditions).	Coastal area, with the highest proportion of people over 60 compared to England.	Social prescription provides patients with practical support in the community in order to respond to their needs. Through the liaison of a trained, non-clinical liaison professional, the “holistic model” is performed to improve the patient’s self-efficacy, the ability to maintain or improve their long-term health.
Kiely *et al.,* 2021	Ireland, Dublin	Protocol for a pragmatic randomised controlled trial	Patients in disadvantaged communities, chronic diseases, the presence of polypharmacy	Disadvantaged area by the population deprivation index	The health professional, linked to primary health care, known as a link worker, provides a friendly prescription to patients, based on knowledge of the resources and services of the local community, in order to improve health outcomes.
Moffatt *et al.,* 2017	Newcastle, UK	Qualitative study	Thirty community people with long-term conditions.	Urban area of high socioeconomic deprivation.	Health professionals refer patients to a professional liaison for non-clinical social prescribing, using voluntary and community sector services, in order to improve patient health.
Munford *et al.*, 2020	Salford, UK	Longitudinal cohort study	Older people living in the community.	Not reported	Patient referral or referral with non-medical sources of support within the community - community assets.

### Description of the studies

The selected studies comprised two randomized controlled trials (RCT), three observational non-controlled before-and-after study, one pragmatic cluster-randomized controlled feasibility trial, one longitudinal cohort study, one protocol for a pragmatic randomized controlled trial, and one qualitative study. Seven studies were conduct in the UK, one in the South Korea, and one in the US.

Four studies, targeted populations with chronic diseases (Elston *et al.*, 2019; Kiely *et al.*, 2021; Loftus *et al.*, 2017; Moffatt *et al.*, 2017), one study focused individuals identified as needing to improve their physical and mental well-being (Howarth *et al.*, 2020), and another study, target participants at risk of loneliness and social isolation (Thomson *et al.*, 2018). The studies were conducted within community settings, including in rural areas (Clifford *et al.*, 2022; Kim *et al.*, 2021) and two in socioeconomically disadvantaged regions (Kiely *et al.*, 2021; Moffatt *et al.*, 2017).

In all studies, referrals to social prescription programs were mainly made by health professionals, particularly in primary health care. In seven articles, a link-worker played a crucial role in bridging the gap between primary care professional, patients, and third sector organizations (Elston *et al.*, 2019; Howarth *et al.*, 2020; Kiely *et al.*, 2021; Kim *et al.*, 2021; Loftus *et al.*, 2017; Moffatt *et al.*, 2017; Thomson *et al.*, 2018).

A total of 4026 participants were involved in these studies. The sample size, age, and sex of the participants are detailed in Table 2. In eight studies, participants were of both sexes, with a higher participation percentage being female. The remaining study included female participants only (Kim *et al.*, 2021).

### Domains of social prescription and its intervention

The most common social prescription activity was arts-related, six of the articles (Clifford *et al.*, 2022; Kim *et al.*, 2021; Loftus *et al.*, 2017; Moffatt *et al.*, 2017; Munford *et al.*, 2020; Thomson *et al.*, 2018). Followed by personal development, five studies (Elston *et al.*, 2019; Kiely *et al.*, 2021; Kim *et al.*, 2021; Loftus *et al.*, 2017; Moffatt *et al.*, 2017) and social interaction four studies (Kiely *et al.*, 2021; Loftus *et al.*, 2017; Moffatt *et al.*, 2017; Munford *et al.*, 2020). Physical activity was reported in three studies (Loftus *et al.*, 2017; Moffatt *et al.*, 2017; Munford *et al.*, 2020) and gardening activity in two studies (Howarth *et al.*, 2020; Kim *et al.*, 2021). Cultural, religious (Munford *et al.*, 2020), and technological activities (Loftus *et al.*, 2017) were each reported in one study.

To obtain a clearer view of the results of this review, social prescription activities were grouped based on the theoretical framework of WHO (2020), as can be seen in Table 4.

**Table 4.** Results tables of social prescription activities

Community resources	Social prescribing activity
Volunteering opportunities	Voluntary organisations; charity, voluntary or community group
Social support	Social clubs, social interaction; self-help group; emotional support and counseling and practical assistance in reaching local health, social; welfare rights services; addiction services; group for elderly, social club, religious group, or church organization
Artistic activities	Arts program activities, craft activities, crochet activities; interactive museum tours; musical interventions; programme the music and dance; choir, photography; arts, music, or singing group
Physical well-being	Fitness activities; gardening activities; exercise classes; physical activity/fitness; weight management, healthy eating; swimming, fishing; sports club, gym, exercise, or dance group
Lifelong learning	Computer courses, personal development; resilience training; chronic disease self-management courses; education group
Housing support	Fall prevention and counseling classes
Financial support	Emotional support and counseling assistance in economic services

The duration of social prescribing interventions ranging on average from ten weeks (Kim *et al.*, 2021; Thomson *et al.*, 2018) to twelve weeks (Clifford *et al.*, 2022; Elston *et al.*, 2019; Howarth *et al.*, 2020; Loftus *et al.*, 2017). In one study, the intervention was longer than 6 weeks (Kiely *et al.*, 2021). And implicitly two studies reported longer intervention periods, 4–14 months (Moffatt *et al.*, 2017), and 18 months (Munford *et al.*, 2020).

Data collection was typically conducted before and after the intervention (Elston *et al.*, 2019; Howarth *et al.*, 2020; Kiely *et al.*, 2021; Kim *et al.*, 2021), with some studies also conducting at multiple points post-intervention (Loftus *et al.*, 2017; Munford *et al.*, 2020; Thomson *et al.*, 2018). In one study, data collection occurred during the intervention (Moffatt *et al.*, 2017).

### Impact on the individual, on the health and care system(s), and community

The results indicate that five studies reported an increase in the physical and psychological well-being of the population (Elston *et al.*, 2019; Howarth *et al.*, 2020; Loftus *et al.*, 2017; Moffatt *et al.*, 2017; Thomson *et al.*, 2018), as well as improved the quality of life (Kiely *et al.*, 2021; Munford *et al.*, 2020). Social prescription was found to decrease isolation (Howarth *et al.*, 2020; Kiely *et al.*, 2021; Loftus *et al.*, 2017; Moffatt *et al.*, 2017) and loneliness (Kim *et al.*, 2021). Health promotion was another, and outcome present three articles (Elston *et al.*, 2019; Kim *et al.*, 2021; Moffatt *et al.*, 2017). Four of the articles assessed the impact of social prescribing on primary health care resources, with one reporting long-term care cost reductions (Munford *et al.*, 2020). However, there was no evidence of fewer visits to primary care. Primary health, decreased polypharmacy, or economic impact in three other studies (Elston *et al.*, 2019; Loftus *et al.*, 2017; Moffatt *et al.*, 2017).

## Discussion

Social prescription activities will create opportunities for health, well-being, and quality of life, a very significant response in preventing disease and promoting the health of older people (Smith *et al.*, 2019), so in the current health scenario our aims to identify the existing social prescription activities for the elderly (≥65 years) in a community context.

### Main findings regarding social prescribing activities

The investigation revealed a variety of social prescribing activities for older people that can be categorized into seven domains. Artistic activities (e.g., arts programs, craft or crochet activities; artistic activities inspired by exhibitions; music; dance; photography; visits to museums, painting, sculpture, choir) were the most frequent domain in social prescription programs, being associated with improved mental health and well-being (Thomson *et al.*, 2018). Ongoing training such as self-care and personal development (e.g., lectures; resilience training; emotional support; chronic illness self-management courses; education groups; counseling to help reach health, social and local services; computer courses), another area that was frequently mentioned was the fact that education for self-care can be effective in improving the patient’s condition to face a variety of chronic diseases (Loftus *et al.*, 2017).

The area of social interaction was the third most present, with social support (e.g., self-help groups; social clubs; elderly groups; fishing); Just as we can insert the domain of volunteering opportunities here (e.g., volunteer or community group; charity), but participants reported significantly higher quality of life in all domains – environmental, psychological, physical, and social (Munford *et al.*, 2020). Physical well-being (e.g., gym; sports club; exercise group; swimming; gardening activities) is a recognized area of health promotion (Moffatt *et al.*, 2017). Finally, the areas of housing support (e.g., fall prevention classes and counseling at home) and financial help (e.g., counseling to help achieve economic services, with practical help), were those with the least examples of activities were found, but which help to achieve significant improvements in health and well-being (Elston *et al.*, 2019).

In comparison with other existing literature, Menhas *et al.* (2023) carried out research in China on social prescription interventions and some of their results are interesting and relevant, namely the topic of E-social prescription, which in the century we live in is increasingly rooted in our daily lives. In this context, we could have activities such as an online health community, telecare services, digital literacy or health trackers (health monitoring and management). Some care would need to be taken with the E-social prescription, since the objective is to connect the person to the community and not isolate them from it, so it could be an intervention to add or complement, for example, in elderly people in rural areas, very isolated and without the possibility of transport.

### Social prescribing framework and interventions

These diverse social prescription activities, initiated by a health professional in primary care, are designed enhance the overall well-being of clients. Referrals can be made by a community nurse, whose role includes promoting self-care and autonomy, understanding their patients, and advocating a salutogenic model of action (Howarth *et al.*, 2020). Following a referral, a link-worker typically liaises with the individual to establish meaningful health and well-being goals and connect them with community resources (Dayson & Bashir, 2014) (Moffatt *et al.*, 2017).

The duration of the social prescription interventions can vary in frequency, duration, and degree of personalization, averaging twelve weeks, but can extend for months. This significant difference observed in health services is also related to the country where social prescription was implemented and the target population, whether it is a broader population, or a specific one, such as based on medical conditions, sociodemographic characteristics, or previous use of healthcare (Morse *et al.*, 2022).

However, no significant difference was observed in the frequency of health services utilization, such as general practice visits or home visits (Loftus *et al.*, 2017). Similarly, no reduction in polypharmacy or economic impact was noted (Elston *et al.*, 2019; Moffatt *et al.*, 2017), although there may be a decrease in long-term care costs (Munford *et al.*, 2020).

### Main findings regarding outcomes

The living context of the elderly, especially in rural areas and post-COVID-19 settings, significantly influences the effectiveness of social prescription interventions. In rural areas, and during post-COVID-19 pandemic era, where isolation, loneliness, and depression were prevalent, social prescription interventions can offer an effective psychological defense for these individuals (Clifford *et al.*, 2022; Kim *et al.*, 2021).

Therefore, these interventions are beneficial for the elderly who are at risk of loneliness and isolation (Thomson *et al.*, 2018), multimorbidity (Kiely *et al.*, 2021) and the vulnerability of physical and mental well-being (Howarth *et al.*, 2020) vulnerability.

Studies incorporating these social prescription activities have reported positive outcome at both intrapersonal and interpersonal levels, including improvement in physical and psychological well-being, self-esteem, quality of life, reduced isolation, and loneliness (Elston *et al.*, 2019; Howarth *et al.*, 2020; Kiely *et al.*, 2021; Kim *et al.*, 2021; Loftus *et al.*, 2017; Moffatt *et al.*, 2017; Munford *et al.*, 2020; Thomson *et al.*, 2018).

There is also an increased social participation (Kim *et al.*, 2021), sense of privilege (Thomson *et al.*, 2018), confidence (Howarth *et al.*, 2020; Moffatt *et al.*, 2017), lessened the impact of chronic diseases (Loftus *et al.*, 2017), and the increase in health promotion (Kim *et al.*, 2021; Moffatt *et al.*, 2017).

## Limitations

Despite a comprehensive research strategy (Table 1), there are some limitations to this review. Firstly, even with the extensive eligibility criteria and the combinations of terms referring to Population (Elderly Person), Concept (Community Prescriptions), and Context (Health Care studies), there may be relevant studies that were not identified.

Additionally, there may be other articles published in scientific databases that we do not have access to, which could have provided valuable data to complementing this review. In future research, it could be beneficial to include terms such as community interventions in the research and later check whether they corresponded to social prescription interventions, as there are still some mixtures between these concepts, being misused and being able to obtain significant articles that refer to social prescription.

Another limitation is the lack of a unanimous definition of social prescription and the absence of social context descriptions in the articles, which could influence the interpretation of the reported results. Despite these limitations, this study suggests the need for further research on social prescribing activities for the elderly population in community settings.

It is also important to highlight another limitation that the included studies were not assessed or weighted for their quality, but only for their relevance to the aim of this review.

## Future recommendations

More qualitative and quantitative research is needed to obtain more robust evidence of social prescription activities for the elderly in a community context. There is still heterogeneity in social prescription models, which makes their investigation difficult, given the type of services and activities referred, characterization of the sample, specifying age, sex, rural or urban context, number of sessions attended, adherence to the service, duration, effects, and dropout rates are important to provide robustness to the investigation (Husk *et al.*, 2020).

Another recommendation would be to evaluate more diverse populations, that is, to carry out investigations in different countries and different health systems, so that there is a more global perspective.

Finally, it should be noted that the involvement of the elderly person themselves, healthcare providers, or caregivers should be involved in future investigations, as they provide a more holistic view of the topic.

## Conclusion

This review aimed to identify the social prescription activities that exist for the elderly in a community context. The primary areas of action identified encompassed artistic, sports, personal development, gardening, social interaction, and technological and cultural activities.

While the concept of social prescription is still evolving, especially outside the UK, its focus on community-based interventions offers potential benefits for the elderly. Social prescription, with focus on individuals within their communities, fosters a more sustainable context, and promotes greater adherence, commitment, and continuity of social prescription activities among the elderly. Primary health care professionals can play a significant role in this process, reinforcing the pertinence of a link-worker.

The potential benefits of social prescribing activities can bring substantial benefits in terms of well-being, quality of life and reducing isolation and loneliness in older people, offer person-centred care, strengthen preventative care, address social determinants of health and social needs, thus promoting healthy ageing. Social prescribing activities aim to advance health and social systems by approaching the person holistically in order to improve their health and well-being, which is crucially important in the wake of the COVID-19 pandemic. It is crucial that future studies not only explore ways to enhance social prescribing for the elderly but also focus, on the implementation of social prescribing programs specifically designed for this demographic.

Furthermore, future research should delve deeper into the various factors (the role of family and caregivers, the socioeconomic context, and the skills of health professionals) that influence social prescription for older people in community contexts. By doing so, we can better understand and optimize the impact of social prescription on the elderly population.

## References

[ref1] Arksey H and O’Malley L (2005) Scoping studies: towards a methodological framework. International Journal of Social Research Methodology: Theory and Practice 8, 19–32.

[ref2] Benedetti TRB , Borges LJ , Petroski EL and Gonçalves LHT (2008) Physical activity and mental health status among elderly people. Revista de Saude Publica 42, 302–307. 18327498

[ref3] Boydell K (2020) The art of social prescription. In Rhodes P (ed), Beyond the Psychology Industry. Cham: Springer, pp. 128–152.

[ref4] Calaf C and González-Vianab A (2021) Herramientas para una orientación comunitaria de la atención primaria: el mejor sustrato para la prescripciónsocial. Formación Médica Continuada En Atención Primaria 28, 21–32.

[ref5] Chatterjee HJ , Camic PM , Lockyer B and Thomson LJM (2018) Non-clinical community interventions: a systematised review of social prescribing schemes. Arts and Health 10, 97–123.

[ref6] Chng NR , Hawkins K , Fitzpatrick B , O’Donnell CA , Mackenzie M , Wyke S and Mercer SW (2021) Implementing social prescribing in primary care in areas of high socioeconomic deprivation: process evaluation of the “deep end” community links worker programme. British Journal of General Practice 71, E912–E920. 10.3399/BJGP.2020.1153 PMC8463130 34019479

[ref7] Clifford AM , Ni Bhriain O , Byrne S , Cheung P-S , Louw Q , Glynn L , Moss H , O’Neill D , Woods CB , Sheikhi A , Gowran RJ , Maher C , Kennelly B , Salsberg J and Thabane L (2022) Music and movement for health: protocol for a pragmatic cluster-randomised feasibility pilot trial of an arts-based programme for the health and wellbeing of older adults. Health Research Board Open Research 5, 1–16. 10.12688/hrbopenres.13535.1 PMC9843089 36726486

[ref8] Comissão económica (2017) Resumo de Políticas. Geneva: Comissão Económica.

[ref9] Dayson C and Bashir N (2014) The Social and Economic Impact of the Rotherham Social Prescribing Pilot: Main Evaluation Report. Sheffield: Centre for Regional Economic and Social Research.

[ref10] Elston J , Gradinger F , Asthana S , Lilley-Woolnough C , Wroe S , Harman H and Byng R (2019) Does a social prescribing “holistic” link-worker for older people with complex, multimorbidity improve well-being and frailty and reduce health and social care use and costs? A 12-month before-and-after evaluation. Primary Health Care Research & Development 20, 1–10. 10.1017/S1463423619000598 PMC6764188 31547895

[ref11] Graybill EM , McMeekin P and Wildman J (2014) Can aging in place be cost effective? A systematic review. PLoS ONE 9, e102705. 25058505 10.1371/journal.pone.0102705 PMC4109953

[ref12] Howarth M , Griffiths A , Silva A and Green R (2020) Social prescribing: a ‘natural’ community-based solution. British Journal of Community Nursing 25, 294–298. 32496851 10.12968/bjcn.2020.25.6.294

[ref13] Husk K , Blockley K , Lovell R , Bethel A , Lang I , Byng R and Garside R (2020) What approaches to social prescribing work, for whom, and in what circumstances? A realist review. Health and Social Care in the Community 28, 309–324. 31502314 10.1111/hsc.12839 PMC7027770

[ref14] INE (2014) Principais tendências demográficas: as últimas décadas. In Famílias nos Censos 2011 Diversidade e Mudança. Lisboa: INE.

[ref15] INE (2020) Projeções de População Residente 2080. Contudo, na Área Metropolitana de Lisboa e no Algarve a População Residente Poderá Aumentar. Lisbon: Statical Portugal.

[ref16] Islam MM (2020) Social prescribing—an effort to apply a common knowledge: impelling forces and challenges. Frontiers in Public Health 8, 515469. 33330299 10.3389/fpubh.2020.515469 PMC7728793

[ref17] Kiely B , Connolly D , Clyne B , Boland F , O’Donnell P , Shea EO and Smith SM (2021) Primary care-based link workers providing social prescribing to improve health and social care outcomes for people with multimorbidity in socially deprived areas (the LinkMM trial): pilot study for a pragmatic randomized controlled trial. Journal of Multimorbidity and Comorbidity 11, 1–9. 10.1177/26335565211017781 PMC8142241 34094992

[ref18] Kim JE , Lee YL , Chung MA , Yoon HJ , Shin DE , Choi JH , Lee S , Kim HK and Nam EW (2021) Effects of social prescribing pilot project for the elderly in rural area of South Korea during COVID-19 pandemic. Health Science Reports 4, 1–11. 10.1002/hsr2.320 PMC8247938 34250272

[ref19] Kyriazis M , Mikellides G , Pantelidakis H , Polycarpou M and Panayiotou B (2021) COVID-19 isolation and risk of death in cyprus elderly people. Frontiers in Medicine 8, 2019–2022. 10.3389/fmed.2021.717692 PMC8365165 34409055

[ref20] Lebrasseur A , Fortin-Bédard N , Lettre J , Raymond E , Bussières EL , Lapierre N , Faieta J , Vincent C , Duchesne L , Ouellet MC , Gagnon E , Tourigny A , Lamontagne MÈ and Routhier F (2021) Impact of the COVID-19 pandemic on older adults: rapid review. JMIR Aging 4, e26474. 33720839 10.2196/26474 PMC8043147

[ref21] Levac D , Colquhoun H and O’Brien KK (2010) Scoping studies: advancing the methodology. Implementation Science 5, 1–9. 20854677 10.1186/1748-5908-5-69 PMC2954944

[ref22] Loftus AM , McCauley F and McCarron MO (2017) Impact of social prescribing on general practice workload and polypharmacy. Public Health 148, 96–101. 28458122 10.1016/j.puhe.2017.03.010

[ref23] MacLeod S , Schwebke K , Hawkins K , Ruiz J , Hoo E and Yeh CS (2018) Need for comprehensive health care quality measures for older adults. Population Health Management 21, 296–302. 29064345 10.1089/pop.2017.0109 PMC6070128

[ref24] Mendes A (2021) Social prescribing in the community. British Journal of Community Nursing 26, 204–205. 33797961 10.12968/bjcn.2021.26.4.204

[ref25] Menhas R , Yang L and Danish Nisar R (2023) Community-based social healthcare practices in China for healthy aging: a social prescription perspective analysis. Frontiers in Public Health 11, 1252157. 37849719 10.3389/fpubh.2023.1252157 PMC10578489

[ref26] Moffatt S , Steer M , Lawson S , Penn L and O’Brien N (2017) Link Worker social prescribing to improve health and well-being for people with long-term conditions: qualitative study of service user perceptions. BMJ Open 7, e015203. 10.1136/bmjopen-2016-015203 PMC5541496 28713072

[ref27] Morley JE and Silver AJ (1995) Nutritional issues in nursing home care. Annals of Internal Medicine 123, 850–859. 7486469 10.7326/0003-4819-123-11-199512010-00008

[ref28] Morse DF , Sandhu S , Mulligan K , Tierney S , Polley M , Chiva Giurca B , Slade S , Dias S , Mahtani KR , Wells L , Wang H , Zhao B , De Figueiredo CEM , Meijs JJ , Nam HK , Lee KH , Wallace C , Elliott M , Mendive JM and Husk K (2022) Global developments in social prescribing. BMJ Global Health 7, e008524. 10.1136/bmjgh-2022-008524 PMC9115027 35577392

[ref29] Morton L , Ferguson M and Baty F (2015) Improving wellbeing and self-efficacy by social prescription. Public Health 129, 286–289. 25744110 10.1016/j.puhe.2014.12.011

[ref30] Munford LA , Panagioti M , Bower P and Skevington SM (2020) Community asset participation and social medicine increases qualities of life. Social Science and Medicine 259, 1–10. 10.1016/j.socscimed.2020.113149 PMC7397510 32603958

[ref31] NHS England and NHS Improvement (2020) Personalised Care Social Prescribing and Community-Based Support Summary Guide. London: NHS England.

[ref32] Ömer Ş , Hasan K and Kübra Ş (2021) The weak ring of the COVID-19 pandemic: the elderly (a literature review). Journal of Geriatric Medicine and Gerontology 7, 1–7.

[ref33] ONLCP (2020) Envelhecimento E Políticas Sociais Em Portugal Que Respostas E Que Futuro? Porto, Portugal: ONLCP.

[ref34] Page MJ , McKenzie JE , Bossuyt PM , Boutron I , Hoffmann TC , Mulrow CD , Shamseer L , Tetzlaff JM , Akl EA , Brennan SE , Chou R , Glanville J , Grimshaw JM , Hróbjartsson A , Lalu MM , Li T , Loder EW , Mayo-Wilson E , McDonald S , … Moher D (2021) The PRISMA 2020 statement: an updated guideline for reporting systematic reviews. The BMJ 372, n71. 33782057 10.1136/bmj.n71 PMC8005924

[ref35] Peixoto N , Lima LCV and Bittar CML (2017) Percepções sobre qualidade de vida entre idosos que participam de uma Universidade Aberta para Maturidade. Acta Scientiarum. Human and Social Sciences 39, 209.

[ref36] Pescheny J , Randhawa G and Pappas Y (2018) Patient uptake and adherence to social prescribing: a qualitative study. Royal College of General Practitioners Open 2, 1–12. 10.3399/bjgpopen18X101598 PMC6189784 30564731

[ref37] Smith TO , Jimoh OF , Cross J , Allan L , Corbett A , Sadler E , Khondoker M , Whitty J , Valderas JM and Fox C (2019) Social prescribing programmes to prevent or delay frailty in community-dwelling older adults. Geriatrics (Switzerland) 4, 4–8. 10.3390/geriatrics4040065 PMC6960851 31783654

[ref38] Thomson LJ , Lockyer B , Camic PM and Chatterjee HJ (2018) Effects of a museum-based social prescription intervention on quantitative measures of psychological wellbeing in older adults. Perspectives in Public Health 138, 28–38. 29130869 10.1177/1757913917737563

[ref39] Tricco AC , Lillie E , Zarin W , O’Brien KK , Colquhoun H , Levac D , Moher, D , Peters MDJ , Horsley T , Weeks L , Hempel S , Akl EA , Chang C , McGowan J , Stewart L , Hartling L , Aldcroft A , Wilson MG , Garritty C and Straus SE (2018) PRISMA extension for scoping reviews (PRISMA-ScR): checklist and explanation. Annals of Internal Medicine 169, 467–473. 30178033 10.7326/M18-0850

[ref40] United Nations Department of Economic and Social Affairs (2019) World Population Ageing 2019. New York: United Nations.

[ref41] World Health Organisation (2022) A Toolkit on How to Implement Social Prescribing. Geneva: WHO.

[ref42] World Health Organization (2015) People-Centred and Integrated Health Services: An Overview of the Evidence. Geneva: WHO.

[ref43] World Health Organization (2023) Progress Report on the United Nations Decade of Healthy Ageing, 2021–2023. Geneva: WHO.

